# Three decades of nearshore surveys reveal long-term patterns in gray whale habitat use, distribution, and abundance in the Northern California Current

**DOI:** 10.1038/s41598-024-59552-z

**Published:** 2024-04-23

**Authors:** Dawn R. Barlow, Craig S. Strong, Leigh G. Torres

**Affiliations:** 1 Geospatial Ecology of Marine Megafauna Lab, Marine Mammal Institute, Department of Fisheries, Wildlife, and Conservation Sciences, Oregon State University, Newport, OR USA; 2 Crescent Coastal Research, Crescent City, CA USA

**Keywords:** Ecology, Biogeography, Biooceanography, Ecological modelling

## Abstract

The nearshore waters of the Northern California Current support an important seasonal foraging ground for Pacific Coast Feeding Group (PCFG) gray whales. We examine gray whale distribution, habitat use, and abundance over 31 years (1992–2022) using standardized nearshore (< 5 km from shore) surveys spanning a large swath of the PCFG foraging range. Specifically, we generated density surface models, which incorporate detection probability into generalized additive models to assess environmental correlates of gray whale distribution and predict abundance over time. We illustrate the importance of coastal upwelling dynamics, whereby increased upwelling only yields higher gray whale density if interspersed with relaxation events, likely because this combination optimizes influx and retention of nutrients to support recruitment and aggregation of gray whale prey. Several habitat features influence gray whale distribution, including substrate, shelf width, prominent capes, and river estuaries. However, the influence of these features differs between regions, revealing heterogeneity in habitat preferences throughout the PCFG foraging range. Predicted gray whale abundance fluctuated throughout our study period, but without clear directional trends, unlike previous abundance estimates based on mark-recapture models. This study highlights the value of long-term monitoring, shedding light on the impacts of variable environmental conditions on an iconic nearshore marine predator.

## Introduction

Throughout the world, humans rely on coastal regions for shipping and commerce, fisheries, industrial development, and increasingly for the development of marine renewable energy^1^. Nearshore environments are therefore coupled social-ecological systems, at the intersection of human and biological productivity^2^. Marine predator species shift their distribution in response to changing ocean conditions, integrating ecological processes across space, time, and trophic levels^3^. For species that live for multiple decades, long-term monitoring is required to assess trends and identify ecological patterns across a broad range of environmental conditions, particularly for long-lived marine species inhabiting dynamic nearshore habitats.

The Northern California Current (NCC) is an eastern boundary current upwelling system that drives enhanced productivity and supports a diverse food web of ecologically and commercially important species^4^. Dynamic oceanographic processes interact with static physical features, likely combining to create key habitat characteristics preferred by coastal predators. Predominant upwelling winds from the north in spring and summer advect nearshore surface waters offshore via Ekman transport, bringing nutrient-rich subsurface water into the photic zone through coastal upwelling. The nearshore region of the NCC (within ~ 5 km of shore^5^) is further shaped by complex bathymetric features including sloping soft bottom substrate, rocky reefs, and kelp forests^6^, creating a rich mosaic of habitat for numerous species of conservation interest, including invertebrates, fish, seabirds, and marine mammals^5^. Additional nutrient influx is provided by the Columbia river plume and large estuarine tidal flows^7,8^, and prominent cape features create areas of recirculation that retain nutrients and bolster recruitment of plankton^9^. Prior research has elucidated how nearshore productivity is maximized when upwelling episodes are interspersed with periods of wind relaxation, which allows phytoplankton to fully utilize the upwelled key nutrients in what is termed the “intermediate upwelling hypothesis”^10^. These relaxation events thereby enhance phytoplankton growth and support larval recruitment and aggregation of crucial forage species^10–13^; however, the interactive effect of upwelling and relaxation on higher trophic level species in nearshore habitats remains poorly understood and largely untested.

At a larger scale, the NCC is also influenced by ocean basin-scale climate patterns. The Pacific decadal oscillation (PDO) can dictate ~ 7–10 year periods of widespread warm or cool conditions throughout the region with consequences across trophic levels^14,15^. Positive phases of the PDO are typically characterized by warmer coastal water temperatures, a more stratified water column, decreased rainfall, and reduced upwelling, whereas periods when PDO is in a negative phase are associated with cooler water temperatures. These phases have ecosystem-wide impacts, with implications for upper trophic level predators; for example, negative PDO phases have been associated with enhanced salmon productivity^16^ and reduced seabird nutritional stress in the eastern pacific^17^. The El Niño southern oscillation (ENSO) is another large-scale climatic pattern with considerable influence in the NCC. Warm ENSO phases, known as El Niño events, are episodic influxes of warmer water resulting in a deeper thermocline and reduced primary productivity in the California Current ecosystem^18^. How these multiple dynamic oceanographic processes interact with static habitat features to influence the distribution and abundance of nearshore predator species in the NCC over decadal time scales warrants further inquiry.

Gray whales (*Eschrichtius robustus*) are an iconic marine predator with a unique subgroup that relies on the dynamic nearshore waters of the NCC. In the northeast Pacific, most gray whales migrate between breeding grounds in the lagoons of Baja California, Mexico, and foraging grounds in the Bering and Chukchi Seas, where they feed on energy-rich benthic amphipods during the summer^19,20^. These whales comprise the Eastern North Pacific stock (ENP), which has made a marked recovery from commercial whaling^21^ when the stock was depleted to less than 5,000 individuals^22^. Nevertheless, while ENP gray whales have rebounded to a high abundance estimate of 28,790 (95% CI 23,620–39,210) in 2015, population size continues to fluctuate, with a most recent estimate of 14,526 (95% CI 13,195–16,040) individuals in 2023^23^. Following the recovery from whaling, the inter-annual variability in ENP population dynamics has been attributed to prey availability and access to arctic foraging grounds^24^. A subgroup of the ENP population shortens their northward migration to forage in the coastal waters between northern California, USA and northern British Columbia, Canada; this sub-group of 212 individuals (SE = 17.9) is known as the Pacific Coast Feeding Group (PCFG)^25^. PCFG gray whales exhibit high inter- and intra-annual site fidelity to this nearshore NCC ecosystem^26^ where they feed on a variety of invertebrate prey including mysid shrimp, crab larvae, amphipods, and cumaceans within a mosaic of habitat including rocky reefs, kelp, sand, boulder, and soft sediment habitats^27,28^. In this study, we focus on the PCFG subgroup.

Investigating the influence of both fine-scale habitat drivers and broad-scale regional climate oscillations on PCFG gray whale distribution could elucidate how they are impacted by both local oceanographic patterns and large-scale environmental change. Climate change and recent marine heatwaves have severely impacted the NCC^29^, with documented reductions in nearshore prey availability^30^ and declining gray whale body condition^31,32^ that indicate population-level consequences of changing environmental conditions. Furthermore, PCFG gray whales are exposed to numerous anthropogenic threats throughout the nearshore waters of the NCC. Interactions with vessels can lead to disruption of foraging^33^, elevated stress hormone levels^34^, or lethal and sub-lethal strike^35^ and overlap with fishing activity can put them at risk of entanglement in fishing gear such as nets or pots^35^. Therefore, improved understanding of their distribution and habitat use patterns is needed to inform subsequent assessments of anthropogenic impacts and shape management priorities accordingly.

While satellite tracking and photo-identification studies of PCFG gray whales have documented home ranges and high-use areas^26,36^, no standardized, coast-wide analysis of PCFG gray whale distribution and abundance in relation to environmental correlates has been conducted. Oscillations in the population dynamics^24,37,38^ and prey availability^20,39,40^ of ENP gray whales relative to environmental variability have been documented; studies of the PCFG have revealed the influence of environmental variability on health^41,42^ and fine-scale patterns in habitat use and prey selection^27,43–46^. Thus, environmental variability influences many aspects of gray whale ecology and vital rates, but knowledge of how static and dynamic habitat characteristics drive PCFG gray whale abundance and distribution within their foraging range is lacking.

In this study, we examine gray whale distribution, habitat use, and abundance between 1992 and 2022 (31 years), and spanning a large portion of the NCC where PCFG gray whales forage^47^ between May and August. This analysis primarily reflects the ecological patterns of the PCFG, rather than the ENP that forage further north during this period: in May, most ENP gray whales are expected to have arrived on their arctic foraging grounds^48^, and PCFG gray whales are known to already be present in the NCC^26^. Nevertheless, we acknowledge the potential inclusion of some late-migrating ENP gray whales during the limited early-season survey effort in our study region. We generate density surface models using standardized line-transect survey data collected with distance sampling methodology, which incorporate distribution and habitat information to compute density estimates. These models enable us to examine the environmental correlates of gray whale habitat use and inter-annual fluctuations in gray whale abundance over the past three decades. This long-term, region-wide habitat analysis lays a foundation for understanding the potential impacts of environmental change on gray whales in the NCC, and can inform management efforts to reduce threats from multiple human activities in the nearshore realm.

## Methods

### Study area

**Figure 1:**
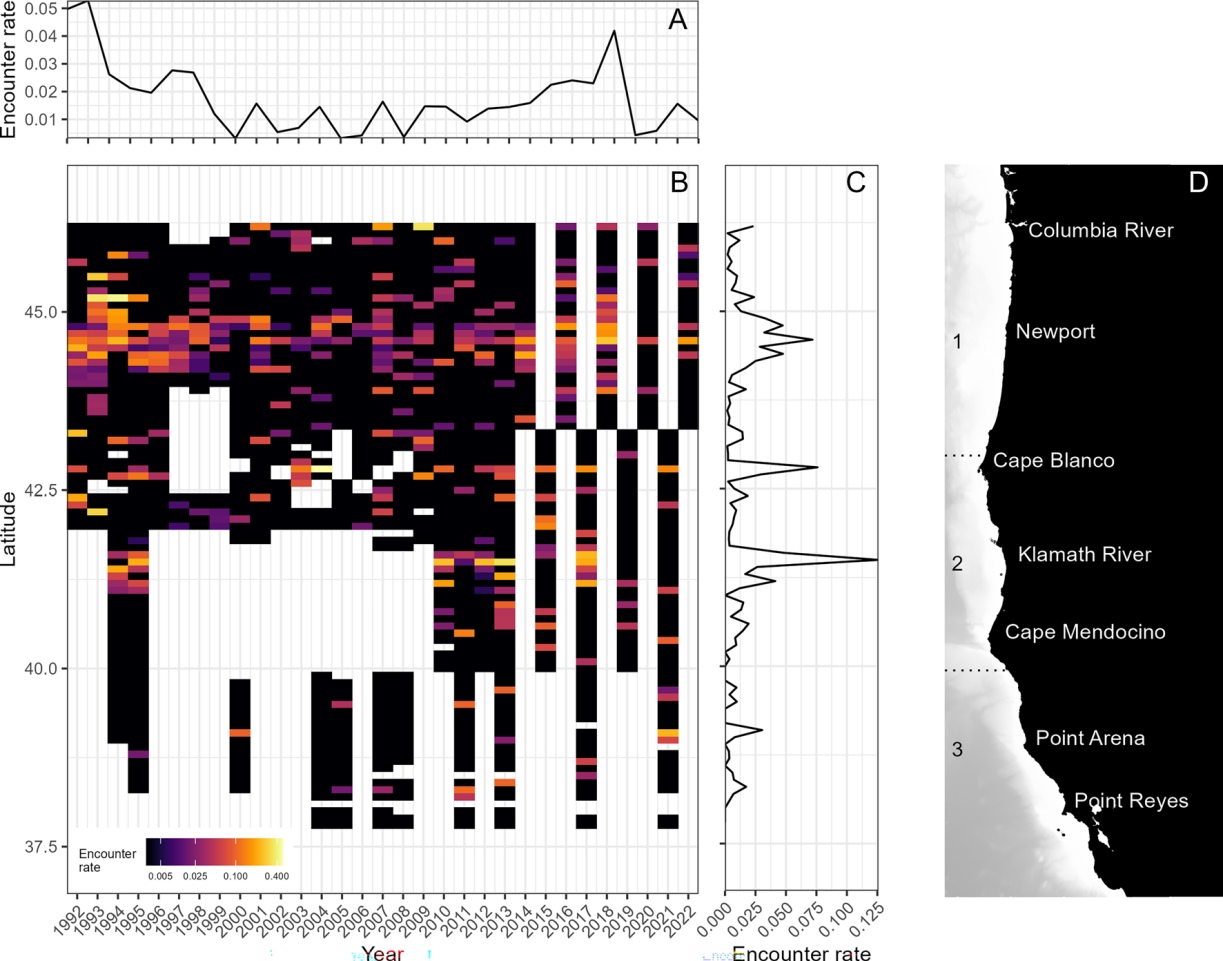
(**A**) Mean gray whale encounter rate (whales/kilometers surveyed) summarized by year, across all latitudes. (**B**) Mean gray whale encounter rate summarized by 1° latitude bin, across all years (note: color ramp visualized on a log scale, sigma = 0.005). White indicates times and locations with no survey effort. (**C**) Mean gray whale encounter rate summarized by year and 1° latitude bin. (**D**) Map of the study area, with region boundaries shown by the dashed lines, and major placenames denoted.

Our survey area extends over a large swath of nearshore waters (< 5 km from shore) along the United States West Coast, between the Columbia River and San Francisco Bay (Fig. 1). This region encompasses three distinct bioregions of the California Current Large Marine Ecosystem^49^ (Supplementary Materials, Fig. S1), including spanning the bioregional division north and south of Cape Blanco. This Cape Blanco landmark divides a moderate upwelling regime along the primarily straight and sandy beaches to the north from strong upwelling centers and a more heterogeneous shoreline from Cape Blanco to Cape Mendocino to the south^50^. Our survey area spans regions 1, 2, and 3, and gray whale encounter rate (Sect. "Field methods") was calculated across all three regions; however, density surface models were only constructed for regions 1 and 2 because gray whale detections in region 3 were too few to construct a robust model (see Sects. "Density surface modeling" and "Survey effort"). Areas of low gray whale encounter rates nevertheless provide useful information on the distribution and range limits of PCFG gray whales in the NCC.

### Field methods

Field research was conducted with the initial objective of monitoring the at-sea abundance of Marbled Murrelets (*Brachyrampus marmoratus*), a seabird of conservation concern, and the sampling scheme was designed accordingly^51–54^. Vessel-based surveys were conducted annually between May and August starting in 1992. The coastline was divided into primary sampling units (PSU), which are contiguous 20 km sections of coast parallel to the shore. Each PSU included four 5 km long “inshore” transect segments running parallel with the coast at four randomly selected distances < 1500 m from shore. Each PSU also included an “offshore” transect, conducted on a diagonal relative to shore from the inshore boundary out to 5 km (north of Coos Bay, Oregon) or 3 km (south of Coos Bay), with a randomized starting point (Supplementary materials, Fig. S2). The randomization of the starting points was established prior to each survey year. In addition to Marbled Murrelets, trained observers recorded all seabird and marine mammal sightings. In this study, we focus on gray whale observations only.

Surveys were conducted aboard a 21-foot Boston Whaler vessel following line-transect, distance sampling methodology^55^. Two observers, one on the port and one on the starboard side of the vessel, scanned between the bow and 90° and − 90° respectively, while the vessel driver maintained a constant survey speed of 10 kt along the transect. Observation conditions, including Beaufort sea state (BSS) and a categorical metric of sightability (ranging from poor to excellent), were recorded and updated continuously while on survey effort. At any gray whale sighting, the perpendicular distance to the trackline was visually estimated to the nearest meter and recorded along with the group size. Observers were trained in distance estimation at the start of each season with weekly calibration testing throughout each year of data collection. Survey methods and study design remained consistent throughout our study period, but survey coverage differed over time; notably, there was a shift to alternating northern and southern areas every other year beginning in 2014 (Fig. 1).

The encounter rate was calculated as the number of gray whale sightings per km of survey effort (n whales / km surveyed). This metric was summarized in three different ways: by year across the full latitudinal range of the study area (37.8°N–46.2°N), by latitude across all years of the study, and by year and 0.1° latitude bin. Encounter rates provide a useful measure of overall gray whale sighting patterns while accounting for effort, and were computed across all three regions in our study area.

### Density surface modeling

#### Detection function

**Figure 2:**
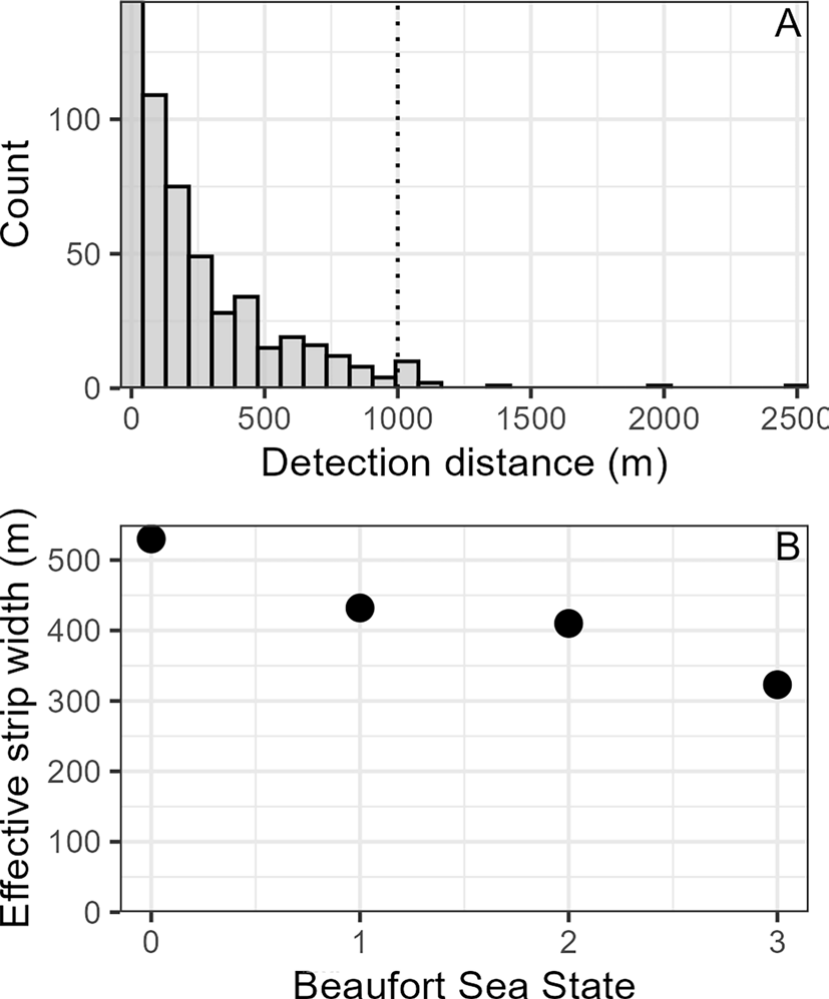
(**A**) Histogram of perpendicular distances from the vessel trackline to gray whale detections. The truncation distance at the 98th percentile is denoted by the black dotted line. (**B**) The effective strip width for detecting gray whales during different Beaufort sea state conditions.

Distance sampling methods use the perpendicular distances between the trackline traveled by the sampler (vessel) and observation (gray whale) to estimate the probability of detection at different distances from the trackline, using a detection function constructed from all distance data^55^. We used the R package ‘Distance’^56^ to fit several candidate detection functions. The truncation distance was set to the 98th percentile of the perpendicular distances, which removed the observations furthest from the trackline that would impact detection function fit while still retaining most of the distance data (Fig. 2). The candidate detection functions were fitted with either no covariates, or with BSS, sightability, or both, and each was tested with both a half-normal and hazard-rate key. Due to the low vessel speed, documented dive times and surfacing intervals of PCFG gray whales^57^, and consistent survey methods throughout the study, we determined that accounting for the availability of gray whales at the surface was not necessary to include in our detection function (i.e., availability = 1). Candidate detection functions were compared using Akaike’s information criterion (AIC). Details on detection function fitting can be found in the Supplementary Materials (Fig. S5, Table S1). The detection function was used to estimate the effective strip width (ESW) under different covariate conditions. In subsequent model fitting, the corresponding ESW was applied to all survey segments according to the conditions at the time.

#### Environmental data

Gray whale distribution and abundance were modeled relative to static and dynamic environmental variables selected to describe the nearshore region of the NCC. In particular, variables assessed included features that may regulate nearshore productivity and retention of upwelled nutrients and thereby potentially influence gray whale foraging opportunities (Table 1, Supplementary Materials, Fig. S3).

**Table 1:** Static and dynamic environmental covariates included in the gray whale density surface models, along with the source of the data and relevant information on how the metrics were computed from the data.

Metric	Description	Source
Depth	Bathymetric depth (m)	GEBCO bathymetry
Distance to coast	Distance to coastline (km)	Open Street Map high resolution coastline
Shelf width	Distance from the coast to the 200 m isobath (km)	GEBCO bathymetry, Open Street Map high resolution coastline; single distance value per latitude applied across longitudes
Distance to cape	Distance and direction to the nearest prominent cape (km)	Distance to the nearest prominent cape; positive values indicate location is south of cape. Cape locations defined as coasline features that differ > 5 km from a 200 m smoothed coastline (high resolution coastline from Open Street Map)
Distance to estuary	Distance to nearest riverine estuary > 300 hectares (km)	Pacific Marine and Estuarine Fish Habitat Partnership
Substrate	Hard or soft benthic substrate	Active Tectonics and Seafloor Mapping Lab, Oregon State University (Oregon), and Seafloor Mapping Lab, California State University Monterey Bay (California)
SST	Sea surface temperature (°C)	Optimal Interpolation SST (0.25 degree resolution), downloaded from ERDDAP
Cumulative CUTI	Daily cumulative upwelling index since the spring transition	Cumulative daily smoothed CUTI since the spring transition index, calculated following Bograd et al. (2009)
Cumulative relaxation	Cumulative number of days where the daily upwelling index value falls below the “relaxation event” threshold	Relaxation events are defined as days when the CUTI value falls below the mean CUTI during the upwelling season (spring transition through end of upwelling). The relaxation threshold is computed separately for each latitude bin, and the mean is calculated across the full study period (1992–2022)

All static environmental layers were computed at a 1 km spatial resolution. Bathymetric depth was obtained from the general bathymetric chart of the oceans (GEBCO, 15 arc-second resolution), and high-resolution coastline information was obtained from the Open Street Map dataset. These two datasets were used to generate continuous layers representing the depth, distance from shore, and shelf width, which was measured as the distance from the coast to the 200 m isobath. Additionally, prominent capes were identified by applying a 200 km continuous smooth to the high-resolution coastline, and locations where the distance between the smoothed and high-resolution coastline was > 5 km were labeled capes. Subsequently, a continuous layer representing distance to the nearest cape was generated for the whole region, with negative values representing locations north of capes and positive values representing locations south of capes. The location and size of river estuaries were obtained from the Pacific Marine and Estuarine Fish Habitat Partnership Estuary Points dataset, which was subset to only include river estuaries > 300 hectares (includes most rivers but excludes small creeks); a continuous layer representing distance to the nearest estuary was generated across the study area. Benthic substrate was classified as either hard or soft based on multibeam sonar mapping conducted, processed, and provided by the Active Tectonics and Seafloor Mapping Lab at Oregon State University for Oregon waters, and the Seafloor Mapping Lab at California State University Monterey Bay for California waters.

Three dynamic environmental variables were examined: sea surface temperature (SST), coastal upwelling, and relaxation. Daily SST data were downloaded from the optimal interpolation SST (OISST, 0.25° resolution) product, which incorporates observations from different platforms (satellites, ships, buoys, and Argo floats) into a regular grid, and spans the full temporal and spatial range of our study. Coastal upwelling and relaxation were obtained from the daily Coastal Upwelling Transport Index (CUTI, 1° latitude bins), which estimates vertical flux off the United States West Coast using regional sea surface height, surface wind stress, and mixed layer depth via regional ocean reanalysis^58^. After a 10-day smoothing filter was applied to the daily CUTI values to reduce the influence of anomalous spikes following previously established methods^59^, a daily cumulative upwelling index was generated for each 1° latitude bin. The cumulative CUTI was used to identify the upwelling season as the period between spring transition index and the end of the upwelling season, following previously established upwelling phenological definitions^4^. Cumulative CUTI was then re-calculated beginning at the spring transition index for subsequent analysis. The mean daily CUTI within the upwelling season was then calculated across the entire study period for each latitude bin, and “relaxation events” were defined as days when the daily CUTI value fell below the mean CUTI during the upwelling season for that latitude bin. Finally, cumulative relaxation was calculated for each day as the cumulative sum of the number of days classified as relaxation events since the spring transition.

Observation conditions and the total number of gray whales were aggregated by survey segment. Environmental data were then extracted at the centroid location of each survey segment for all static layers and SST; cumulative CUTI and cumulative relaxation were assigned to segments by date and latitude bin. While inshore segments were 5 km in length, the offshore segments varied in length due to the sampling scheme; segment length was accounted for in model fitting.

Our study area spanned parts of three established bioregions of the California Current Large Marine Ecosystem^49^ (Supplementary Materials, Fig. S1). We fit spatial models separately for each region to account for documented regional oceanographic differences that may influence gray whale habitat use and foraging patterns in distinct ways.

#### Spatial model fitting

Density surface models (DSM) are an approach for obtaining spatially explicit species abundance and density estimates, by spatially modeling species occurrence patterns while correcting for detection probability under different observation conditions^60^. DSMs consist of a two-stage modeling process. Once the detection function is fit to the distance sampling data, a spatial model is constructed using species count per survey segment as the response variable, correcting for the ESW of each segment as an offset. The spatial component of the DSMs were fit for each region using generalized additive models (GAM), which are semi-parametric regression models that can account for non-linear relationships between predictor and response variables using smoothing functions^61^. For each region, a GAM was fit with a quasi-Poisson distribution and a log link function. The response variable was the number of gray whales observed per survey segment, and predictor variables included smoothed terms for depth, distance to coast, shelf width, distance to cape, distance to river estuary, and SST. Distance to cape was not included for region 1, where few prominent capes exist, and the inclusion of this term creates spurious ecological inferences. Substrate was included as a parametric term (categorized as either hard or soft based on the predominant value of each survey segment). Additionally, we included a smoothed interaction term between cumulative CUTI and cumulative relaxation. All smoothed terms were fitted with a restricted maximum likelihood (REML) smoothing parameter estimation method. To reduce model overfitting, smoothed terms were restricted by setting the number of knots used for basis construction, which effectively control the degree of smoothing allowed, to k = 5, and the smoothed interaction term between cumulative CUTI and cumulative relaxation was set to k = 15. Variable selection was conducted with a shrinkage approach implemented, which adds an extra penalty to each smoother and penalizes non-significant variables to zero^62^. DSM model fit was evaluated using percent deviance explained and by visualizing quantile plots and the distribution of the residuals, as well as by comparing predicted abundances to the raw encounter rate data. All DSMs were implemented in the ‘dsm’ package in R^63^.

### Assessment of long-term patterns in gray whale abundance

The DSMs were used to predict daily gray whale abundance across a 5 km grid generated between the coastline and 5 km from shore. Daily predictions were produced between 15 May and 30 August of each year for each region, which reflects both the timeframe when surveys were conducted and when PCFG gray whales are expected to be on their foraging grounds^48^; we recognize that gray whales likely move within and outside of our study area during this time. The daily scale for prediction was chosen to capture the temporal variability in our dynamic predictor variables of interest (SST, upwelling, and relaxation). Given that survey effort was variable between years across the study area, including gaps in effort for some areas in certain years (Fig. S4), we took steps to mitigate the potential impact of predicting gray whale abundance for times or locations not represented in the training data. As predictions to periods with non-analogous conditions in the model training data (i.e., environmental conditions never measured during surveys) can lead to unrealistic results^64^, days with high extrapolation by the dynamic predictor variables (SST, cumulative CUTI, cumulative relaxation) were removed from further analysis. This was done using the extrapolation detection (ExDet) tool in the R package ‘dsmextra’^65^, by removing days containing grid cells with ExDet > 1.15 or < − 0.15. After extrapolated days were removed, the mean gray whale abundance was calculated for each grid cell across all predictions and mapped spatially, along with the corresponding uncertainty (coefficient of variation, CV), to identify long-term hotspots in gray whale abundance throughout the entire study area and period.

Daily predicted gray whale abundance was summed across each region, producing a regional daily abundance estimate. These daily predictions were visualized to investigate seasonal patterns in predicted gray whale abundance within the survey period for each year. Then, the mean predicted daily abundance and associated uncertainty was calculated for each study year to obtain a single abundance estimate per year, enabling us to examine long-term inter-annual fluctuations in gray whale abundance for each region. These annual predicted abundances were compared to the annual abundance estimates produced for PCFG gray whales using mark-recapture abundance modeling with individual photo-identification data^25^.

Predicted annual gray whale abundance was compared to long-term, ocean basin-scale oscillations, namely the PDO and the Multivariate ENSO Index (MEI). The timeseries of gray whale abundance in each region were visually compared to the PDO and MEI timeseries. Then, both PDO and MEI were summarized during the spring (February-May) and summer (May–August) periods of each year of the study, and timeseries cross-correlations were conducted using the ‘stats’ package in R to assess whether lagged relationships between these ocean basin-scale patterns and gray whale abundance in each region occurred (up to 10-year lagged relationships).

Linear regression models were run to examine the relationship between gray whale abundance and mean spring and summer PDO and MEI at the time lag determined to be most significant by the timeseries cross-correlations. Finally, we assessed the relationship between gray whale abundance and variability in PDO and MEI over the preceding 1–5 years (measured using standard deviation, sd), and likewise assessed the significance of the relationship using linear regression models. All analyses were conducted in R version 3.6.1^66^.

## Results

### Survey effort

Between 1992 and 2022, the completed transect segments comprised 55,346.2 km of survey effort. Effort varied between years, particularly since 2014 when the survey design shifted to alternating between northern and southern areas every other year. However, across the full study period, survey effort was comprehensive and generally consistent (Supplementary Materials, Fig. S4).

The survey dataset contained 647 gray whale observations, totaling 792 individuals. Encounter rate varied across years and latitude, with coastal areas off Newport, Cape Blanco, and the Klamath River emerging as areas of consistent high encounter rates despite inter-annual fluctuations across the study area (Fig. 1). Observations from all regions were used to fit the detection function; however, very low gray whale encounter rates in region 3 (Fig. 1) meant that it was not possible to generate a robust DSM for region 3. Therefore, the DSMs and all subsequent analyses were limited to regions 1 and 2, spanning between the Columbia River to the north and Cape Mendocino to the south.

### Density surface models

Of the 647 gray whale observations, 528 had associated distance estimates, and were used to fit the detection function (i.e., there were 119 instances where observers did not record distance in the field; these observations were not included in the construction of the detection function, but were included in the DSMs). The truncation distance (98th percentile) was set to 1000 m. The selected detection function was fitted with a half-normal key and included BSS as a covariate (Table S1, Fig. S5). The ESW ranged between 323 and 530 m, depending on BSS conditions (Fig. 2, Table S1). No gray whale observations were made in BSS 4 or above; survey segments with BSS 4 were therefore removed as the detection function could not be applied to estimate the ESW. It should be noted, however, that only 0.68% of survey segments took place in BSS 4.

**Figure 3:**
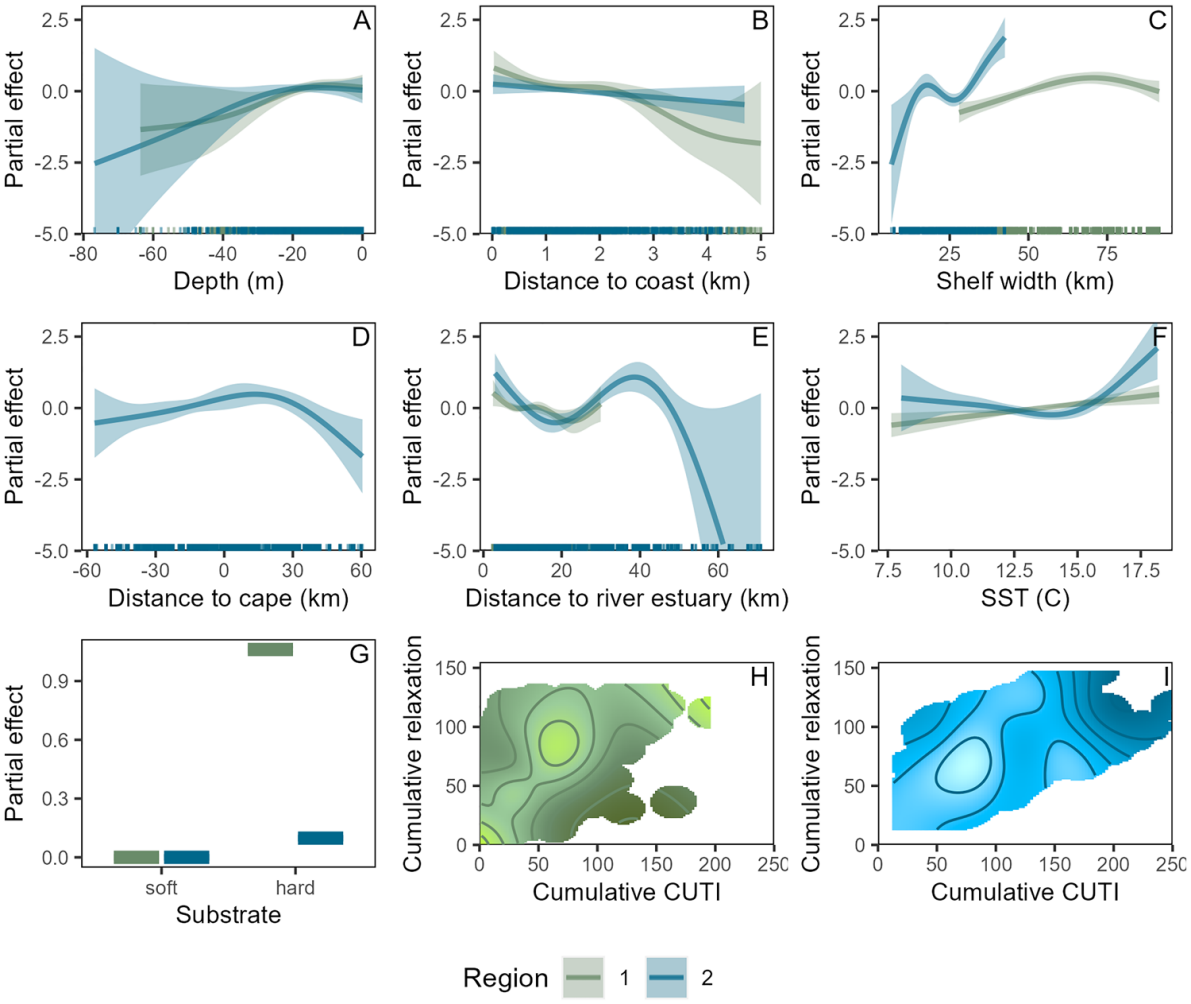
Partial response plots illustrating the functional relationships identified by the density surface models for region 1 (green) and region 2 (blue). (**A**–**F**) functional response curves for smoothed predictor variables. (**G**) functional response for categorical predictor variable. (**H**) functional response for the interactive effect of cumulative CUTI and cumulative relaxation in region 1, with light green coloration representing a strong positive partial effect and dark green representing a strong negative partial effect. (**I**) functional response for the interactive effect of cumulative CUTI and cumulative relaxation in region 2, with light blue coloration representing a strong positive partial effect and dark blue representing a strong negative partial effect. In H and I, areas with no color represent environmental conditions that did not occur in the dataset.

The DSM for region 1 had a deviance explained of 17.8%. Significant predictors in the model included distance to coast, shelf width, benthic substrate, SST, and the interaction between cumulative CUTI and cumulative relaxation (Table 2). Higher gray whale abundances were associated with nearshore waters, greater shelf width, hard bottom substrate, and warmer SST. The highly significant interactive effect of cumulative CUTI and cumulative relaxation revealed an optimal combination whereby increased cumulative CUTI only had a positive effect on gray whale abundance if it coincided with greater cumulative relaxation (Fig. 3).

**Table 2:** Gray whale density surface model performance metrics and predictor variable contribution for region 1 and region 2.

	Metric	Region 1	Region 2
	Deviance explained	17.8%	23.4%
Predictor variable significance in model (*p*-value)	s(depth)	0.102	0.204
	s(distance to coast)	0.006 **	0.115
	s(shelf width)	1.32 × 10^–5^ ***	1.12 × 10^–8^***
	s(distance to cape)	N/A	0.010 *
	s(distance to estuary)	0.050	2.50 × 10^–7^ ***
	Substrate	2.81 × 10^–12^ ***	0.709
	s(SST)	0.003 **	2.40 × 10^–5^ ***
	s(cumulative CUTI, cumulative relaxation)	1.59 × 10^–11^ ***	4.21 × 10^–5^ ***

Overall model performance is measured by the deviance explained. Significant predictor variables are denoted by the asterisks (*) associated with their *p*-value in the model, with the number of asterisks denoting the significance level.

The DSM for region 2 had a deviance explained of 23.4%. Significant predictors included shelf width, distance to cape, distance to estuary, SST, and the interaction between cumulative CUTI and cumulative relaxation (Table 2). Higher gray whale abundances were associated with greater shelf width, areas near estuaries and slightly south of prominent capes, and warmer SST. The interactive effect between cumulative CUTI and cumulative relaxation revealed an optimal combination of moderate upwelling and moderate relaxation, with very high cumulative CUTI or relaxation leading to lower gray whale abundance (Fig. 3).

### Gray whale abundance over time

**Figure 4:**
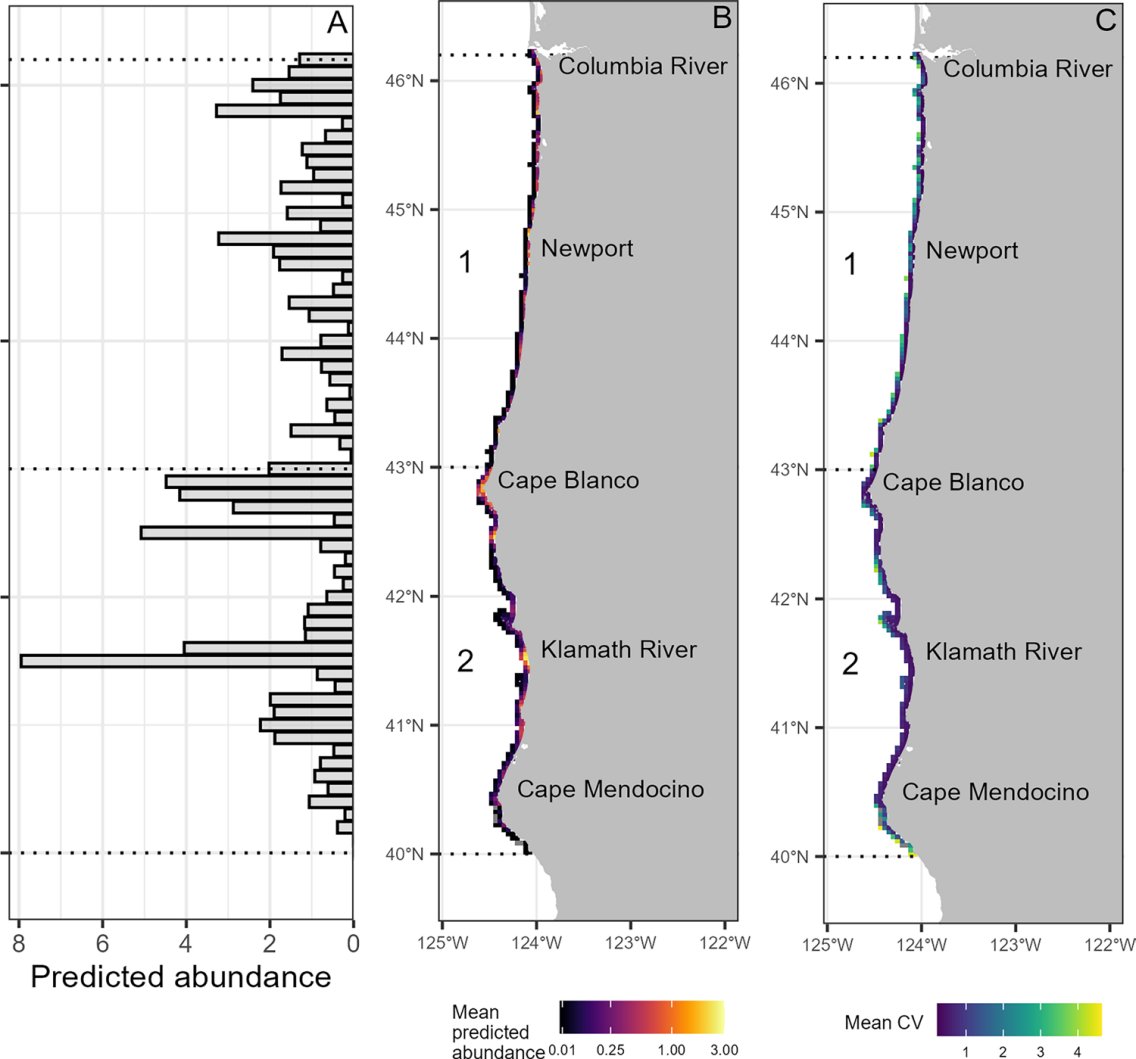
(**A**) Mean predicted gray whale abundance per 1° latitude bin across the entire study period (1992–2022). (**B**) Mean predicted daily gray whale abundance per 5 km grid cell, illustrating fine-scale spatial predictions produced by the density surface models for each region. Note that the color ramp is visualized on a log scale (sigma = 0.1), but true predicted abundance values are displayed. Geographic reference points are denoted in black text. Density surface models were fit and predicted separately for region 1 and region 2, denoted by the dashed black lines. (**C**) Mean associated uncertainty (coefficient of variation, CV) per 5 km grid cell across the entire study period.

The mean gray whale abundance map across the full study period revealed consistent abundance hotspots off the mouth of the Klamath River, near Cape Blanco, and in the nearshore waters extending north and south of Newport (Fig. 4). Areas with high encounter rates observed in the raw survey data (Fig. 1c) were in general agreement with areas of high abundance predicted by the models (Fig. 4a).

**Figure 5:**
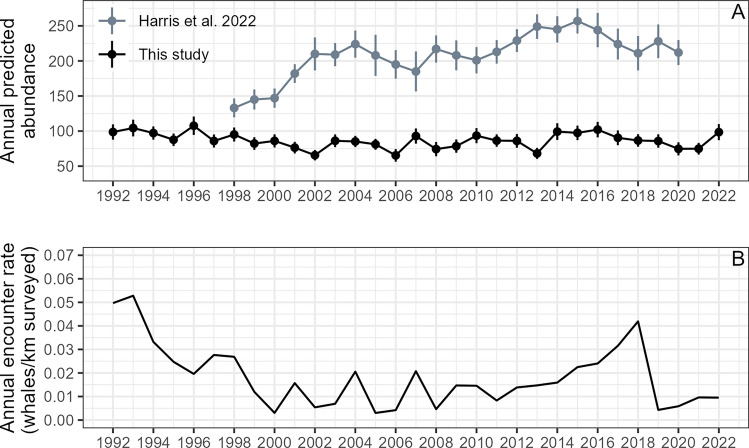
(**A**) Annual mean of the daily predicted abundance estimates summed across regions 1 and 2 of our study area between the Columbia River and Cape Mendocino (40° N–46.2° N) in black, annual abundance estimates for the entire PCFG range based on a mark-recapture model built with photo-identification data across the entire PCFG range between northern California and northern British Columbia (41° N–52° N)^25^ in gray. Confidence intervals shown by the bars represent the standard error around annual estimates. (**B**) Annual encounter rate (number of gray whales/km surveyed) summarized across regions 1 and 2 for each year.

**Figure 6:**
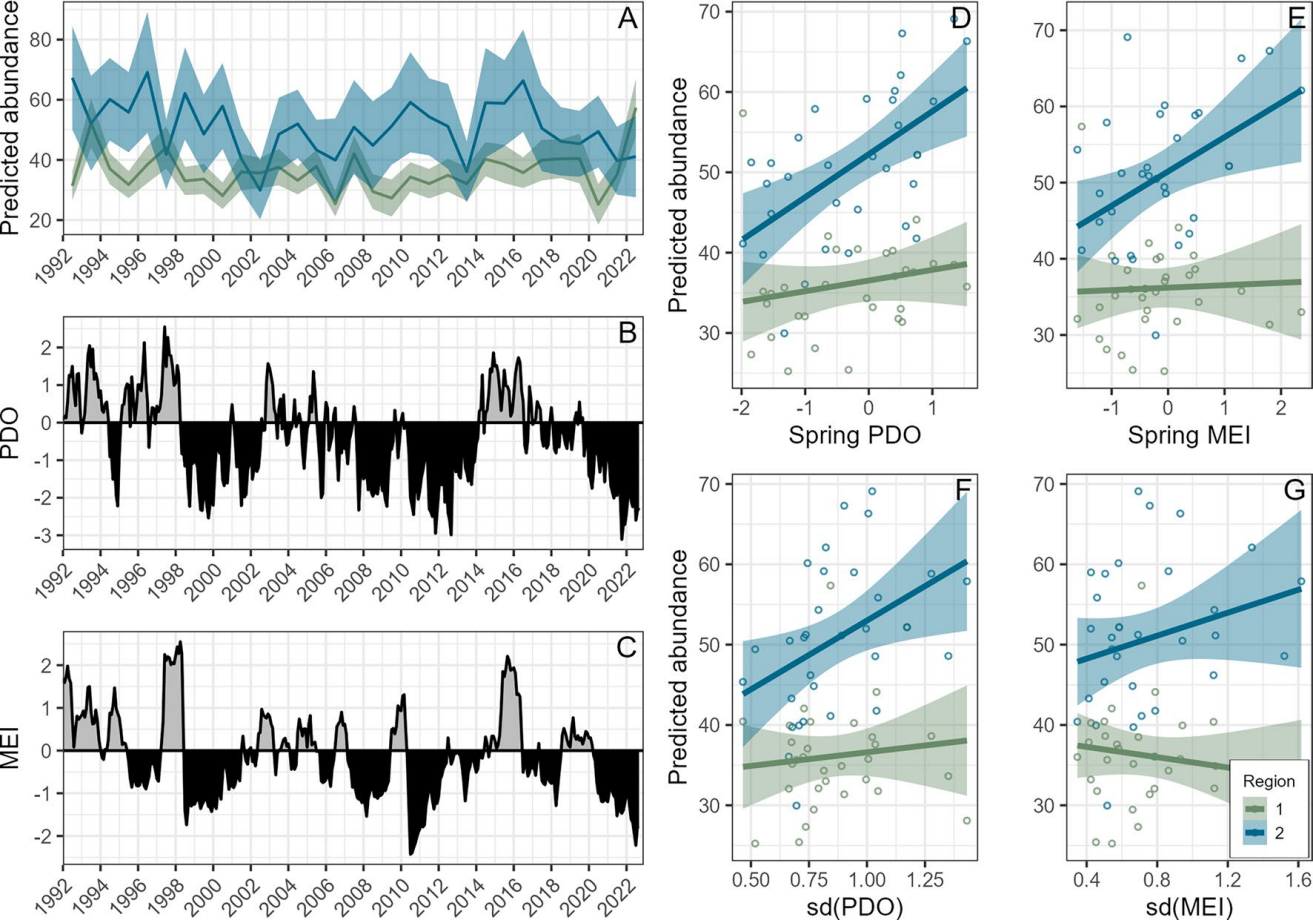
(**A**) Annual mean of the daily predicted gray whale abundance estimates for region 1 (green) and region 2 (blue). (**B**) Pacific decadal oscillation (PDO) index values across the study period. (**C**) Multivariate ENSO index (MEI) values across the study period. (**D**) Linear relationships between predicted annual gray whale abundance and spring PDO in region 1 (green) and region 2 (blue). (**E**) Linear relationships between predicted annual gray whale abundance and spring MEI in region 1 (green) and region 2 (blue). (**F**) Linear relationships between predicted annual gray whale abundance and sd(PDO) over the preceding three years in region 1 (green) and region 2 (blue). (**G**) Linear relationships between predicted annual gray whale abundance and sd(MEI) over the preceding three years in region 1 (green) and region 2 (blue).

Daily predicted abundances across each region showed some seasonal variation within each year, particularly earlier in the season (Supplementary Materials, Fig. S7). The mean annual predicted abundances did show inter-annual variability (Fig. 5), which was at times coincident and at other times divergent between regions (Fig. 6). The total abundance across the entire study area likewise showed inter-annual variability, however, no apparent long-term increasing or decreasing trends in abundance were evident. This result stands in contrast to the abundance estimates computed using mark-recapture models from photo-identification data across the entire PCFG range between northern California and northern British Columbia (41°N–52°N)^25^, which show a generally increasing trend since 1998 when estimates started, followed by a decrease in recent years (Fig. 5). Both studies showed maxima in abundance between 2014 and 2016 (Fig. 5).

While no clear or significant relationship was evident between predicted gray whale abundance and ocean basin-scale climate oscillations in region 1 (Fig. 6; Supplementary Materials, Fig. S8), predicted annual abundance in region 2 was most correlated with spring PDO and MEI within the same year (Supplementary Materials, Fig. S8), displaying a significant positive linear relationship for both PDO (R^2^ = 0.328, F_(1,29)_ = 14.17, *p* < 0.001) and MEI (R^2^ = 0.192, F_(1,29)_ = 6.925, *p* = 0.013) (Fig. 6). There was also a significant positive relationship between predicted abundance and sd(PDO) over the preceding three years for region 2 (R^2^ = 0.171, F_(1,29)_ = 6.003, *p* = 0.020; Fig. 6; Supplementary Materials, Fig. S9, Table S2).

## Discussion

We present the first region-wide gray whale habitat models and corresponding abundance estimates for PCFG gray whales between the Columbia River, Oregon, and Cape Mendocino, California. Our results indicate multiple long-term, stable hotspots of gray whale habitat (Fig. 4), which can be predicted by certain static physical features such as rocky reefs and prominent capes (Fig. 3). Despite these areas of consistent gray whale habitat, PCFG gray whale abundance has fluctuated over the past three decades, with diverging patterns between regions of the NCC (Fig. 6). The dynamic interactive effect of upwelling and relaxation was a strong predictor of gray whale abundance and may therefore be a critical driver of distribution patterns, likely due to their combined role in influencing gray whale foraging opportunities in the nearshore NCC through a crucial combination of “enough but not too much” upwelling or relaxation to enable optimal recruitment and retention of their invertebrate prey. Unraveling these relationships between both static and dynamic environmental drivers and gray whale distribution and abundance over the past three decades not only sheds new light on the ecology of the PCFG, but also lays a foundation for understanding what the future might hold for the nearshore waters of the NCC as the ecosystem contends with impacts global climate change^67^ and multiple anthropogenic pressures^1^.

All our DSMs examining the functional relationships between gray whale distribution patterns and habitat features incorporate the probability of detecting gray whales given the observation conditions at the time. We note that this is the first detection function for gray whales to our knowledge, which can be applied to other surveys conducted in comparable small research vessels. In both region 1 and region 2, the models revealed a positive relationship between gray whale abundance and shelf width (Fig. 3, Table 2). Areas where this distance is greater represent relatively shallow banks extending further offshore, which can generate countercurrents that recirculate water toward the coast, creating areas of locally elevated primary productivity^68,69^. Distance to capes was significant the region 2 models, likely because prominent capes are physical structures along windy coastlines that impact current circulation, and the lee of cape and headland features tend to be areas of enhanced plankton recruitment and settlement^9,70^. Notably, region 2 is characterized by the very prominent Cape Blanco, which is a long-term gray whale abundance hotspot (Fig. 4).

In region 1, hard bottom substrate had a strong positive relationship with gray whale abundance, despite comprising only 5.29% of the benthos across the region. Much of the hard bottom substrate in region 1 occurs in the Newport area (Supplementary Materials, Fig. S3), an apparent hotspot revealed in both the encounter rates (Fig. 1) and model predictions (Fig. 4). Rocky reefs support kelp forests that aggregate epibenthic zooplankton, and gray whales are known to feed in this habitat along the Oregon Coast^46^. Interestingly, region 2 had no significant influence of substrate type, yet distance to estuary was significant. Riverine estuaries are known to play a key role in nutrient cycling in the coastal NCC, with a stronger influence in areas with fewer large coastal watersheds such as region 2 in our study area^7^. Notably, region 2 showed consistent use of waters near the mouth of the Klamath River, corroborating other studies documenting gray whales using this area for benthic foraging in soft sediment habitat^28,71,72^. Taken together, these patterns emphasize the importance of static habitat features (i.e., benthic substrate, shelf width, capes, and estuaries) that influence nearshore retention and recirculation dynamics that enhance nearshore productivity, which in turn yield gray whale foraging opportunities in the NCC.

The interaction between upwelling and relaxation was a significant predictor of gray whale abundance, with a particularly strong effect in region 1 (Fig. 3, Table 2). The functional relationship revealed that more upwelling alone is not necessarily better; rather, an intermediate combination of upwelling accumulation in association with sufficient relaxation events was most beneficial. This pattern supports the intermediate upwelling hypothesis, which posits that input and retention of key nutrients is facilitated by the alternation of upwelling and relaxation periods^10^. This concept has been illustrated in pelagic environments, whereby variability in upwelling-favorable wind facilitates phytoplankton growth, increased zooplankton biomass, and fish recruitment^11,73^. Furthermore, pulsed upwelling events spur the aggregation of key prey species such as krill and forage fish^13^. Here, we illustrate that intermediate upwelling is likewise crucial in nearshore waters of the NCC during the spring and summer to facilitate foraging on epibenthic invertebrate prey by a large marine predator, the gray whale. These findings align with a recent study that investigated the environmental drivers of the zooplankton prey of PCFG gray whales, which also found that upwelling and relaxation are both needed, further supporting the intermediate upwelling hypothesis^74^. The significant positive relationship between gray whale abundance and SST is at first counterintuitive; however, elevated SST on a daily timescale may be a secondary signal of relaxation, lending further evidence to the importance of intermittent upwelling and relaxation for nearshore retention and gray whale foraging. It should also be noted that the importance of relaxation is stronger in region 1; given the lack of prominent capes along Oregon’s central and northern coastline (Supplementary Materials, Fig. S3), this may indicate that relaxation is even more important for retention of upwelled nutrients in areas where there are no prominent capes to drive local recirculation during upwelling conditions.

Predicted PCFG gray whale abundance varied inter-annually, and the fluctuations in abundance were often asynchronous between region 1 and region 2 (Fig. 6). However, the variability in the combined abundance estimates produced by our regional DSMs was much less than the variability in mark-recapture abundance estimates produced from individual photo-identification data across a larger study extent^25^, which generally show a strong increase between 1996 and 2015, followed by a decreasing trend in recent years (Fig. 5). The lower abundance estimate from the DSMs was expected because our study region does not cover the full PCFG range as does^25^. Therefore, it is unsurprising that the absolute abundance estimates differ. The inter-annual trends in abundance estimates derived by the mark-recapture approach that are not reflected in our DSM abundance estimates could be attributed to occupancy fluctuations in the portion of the PCFG range not assessed in our study that extends north from the Columbia River to Vancouver Island, Canada. However, this explanation alone seems unlikely, considering the magnitude of the difference in the patterns, and given that individual gray whales move throughout the study area over the course of each survey year^36^. Photo-identification data collection for the mark-recapture analysis is more spatially and temporally variable from one year to the next across the PCFG range, with spatially and temporally discrete pockets in photo sampling effort^25^, compared to the standardized nearshore survey effort underpinning this study (Supplementary Materials, Fig. S4). While the mark-recapture abundance estimates may be better able to capture changes in recruitment to the PCFG (whether via calf production or recruitment from the broader ENP population), and movement of individuals, this approach is sensitive to unequal sampling effort and does not take into account changes in habitat. In contrast, DSM abundance estimates do not account for site fidelity of individuals, but are meant to reflect the capacity of the habitat to support the population in a particular place and time, and how habitat quality varies throughout their range and between years (it should be acknowledged, however, that they do not consider changes in habitat preferences over time). Therefore, the two methods provide estimates of annual gray whale abundance throughout the PCFG range that differ, due to fundamental differences in the methodological approaches, both worthy of consideration when evaluating population dynamics.

Our study period spanned both positive and negative phases of the PDO and ENSO (Fig. 6), enabling us to explore potential relationships between gray whale abundance and these broad-scale patterns. In the nearshore environment, ocean basin-scale oscillations are known to impact different species in complex ways, likely mediated by key differences in foraging^14,75^. PCFG gray whales show different feeding behaviors and preferred prey items across their range^27,28,45^, and we observed contrasting patterns in the relationship between gray whale abundance and ocean basin-scale oscillations between regions (Fig. 6). In region 2, particularly in the high-use area near the mouth of the Klamath River, gray whales have been documented feeding benthically on invertebrate prey such as cumaceans and amphipods in soft bottom sediment^28,71,76^. The positive relationship between gray whale abundance and both MEI and PDO, and particularly the positive relationship between gray whale abundance and sd(PDO) in region 2 (Fig. 6), corroborate what has been found for other benthic predators in the California Current. Namely, kelp greenling (*Hexagrammos decagrammus*) growth is greatest when ocean temperatures oscillate between cold conditions, which maximize recruitment of benthic invertebrate prey, and warm conditions, which maximize prey growth^14^. In region 1, gray whales are known to feed regularly on epibenthic or pelagic zooplankton, such as mysids, that aggregate in the water column around rocky reefs and kelp beds^30,46,77,78^. For black rockfish (*Sebastes melanops*), another nearshore predator that feeds in the water column around rocky reefs in the California Current, growth was maximized under cool ocean basin-scale conditions (e.g., negative PDO). We did not observe a relationship between cool ocean basin-scale temperatures and elevated gray whale abundance in region 1. Nonetheless, these possible differences in target prey types and their associated life histories may partially explain why the relationships between gray whale abundance and ocean basin-scale oscillations differed between regions (Fig. 6). Additionally, it is possible that PCFG gray whale distribution is compressed during warm ocean basin-scale conditions, or that they may face increased competition with other nearshore predators during cool conditions. The PCFG foraging grounds span a large latitudinal range, covering a diversity of habitat characteristics and prey types. These features likely relate to individual foraging strategies, site fidelity, and movement patterns among PCFG gray whales, with implications for how variability in environmental conditions and prey availability will impact PCFG gray whales in different portions of their range.

The future of eastern boundary current upwelling ecosystems such as the NCC is uncertain, as global climate change is anticipated to drive physical changes in the regional oceanography. Namely, alongshore winds may increase, yielding increased coastal upwelling^79,80^. However, a poleward shift in these upwelling systems will likely lead to long-term changes in the intensity, location, and seasonality of upwelling-favorable winds, with intensification in poleward regions but weakening in equatorward areas^81^. Another projected change is stronger temperature gradients between inshore and offshore areas, and vertically within the water column^67^. What these opposing physical forces will mean for primary productivity and species community structure remains to be seen. In the case of gray whales that rely on nearshore foraging grounds in the NCC, the intensification of upwelling in northern regions may threaten the delicate balance of upwelling and relaxation that provides predictable and accessible prey aggregated near shore. Gray whales will be forced to contend with these environmental changes alongside existing anthropogenic pressures they face in nearshore waters from human impacts such as vessel disturbance^33^, entanglement and vessel strike risk^35^, and ocean noise^34^. Multiple stressors impact the health of PCFG gray whales^82^, emphasizing the need to assess cumulative effects of both environmental and anthropogenic impacts on their foraging grounds to inform management decisions. Continued, long-term data collection can provide valuable context for understanding past and present patterns, and guiding conservation efforts into the future.

### Supplementary Information

Supplementary Information.

## Data Availability

The datasets used in this study are available from the corresponding author upon reasonable request.
