# Supplementary material for: Three decades of nearshore surveys reveal long-term patterns in gray whale habitat use, distribution, and abundance in the Northern California Current
Source: Sci Rep. 2024 Apr 23;14:9352. doi: 10.1038/s41598-024-59552-z (PMC11039675; doi:10.1038/s41598-024-59552-z)
Supplement: Supplementary file 1 — Supplementary Information. [file 41598_2024_59552_MOESM1_ESM.pdf]

Three decades of nearshore surveys reveal long-term patterns in gray whale habitat use, distribution, and abundance in the Northern California Current

Dawn R. Barlow[1]*, Craig S. Strong[2], Leigh G. Torres[1]

[1]Geospatial Ecology of Marine Megafauna Lab, Marine Mammal Institute, Department of Fisheries, Wildlife, and Conservation Sciences, Oregon State University, Newport, Oregon, USA

[2]Crescent Coastal Research, Crescent City, California, USA

*dawn.barlow@oregonstate.edu

SUPPLEMENTARY MATERIALS

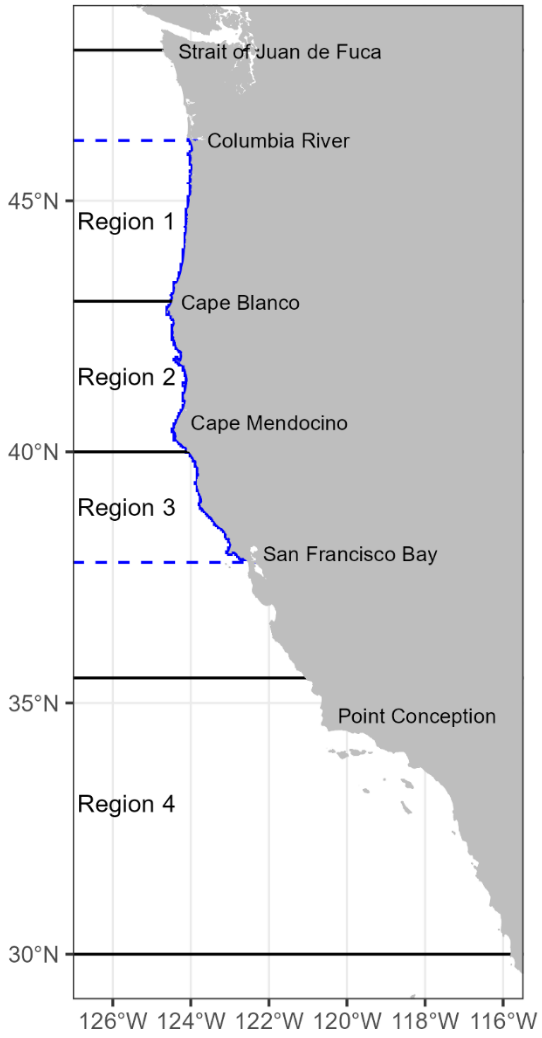

Figure S1. Bioregions of the California Current Large Marine Ecosystem (Schroeder et al. 2022). The survey area for this study is shown in the blue polygon, with study area latitudinal boundaries denoted by the dashed blue lines.

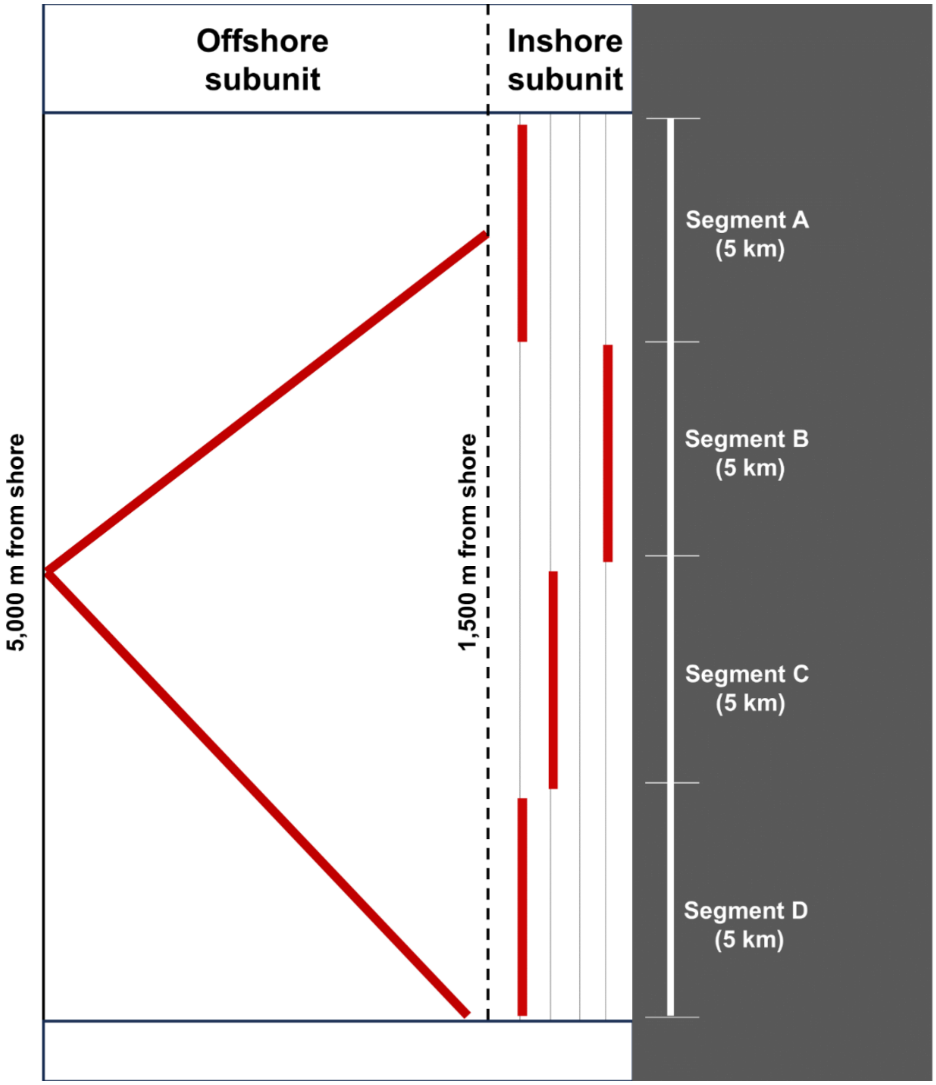

Figure S2. Schematic of the sampling scheme within a primary sampling unit (PSU). The shoreline is denoted by the dark gray. Survey segments are illustrated as the red lines, in both the inshore and offshore subunits.

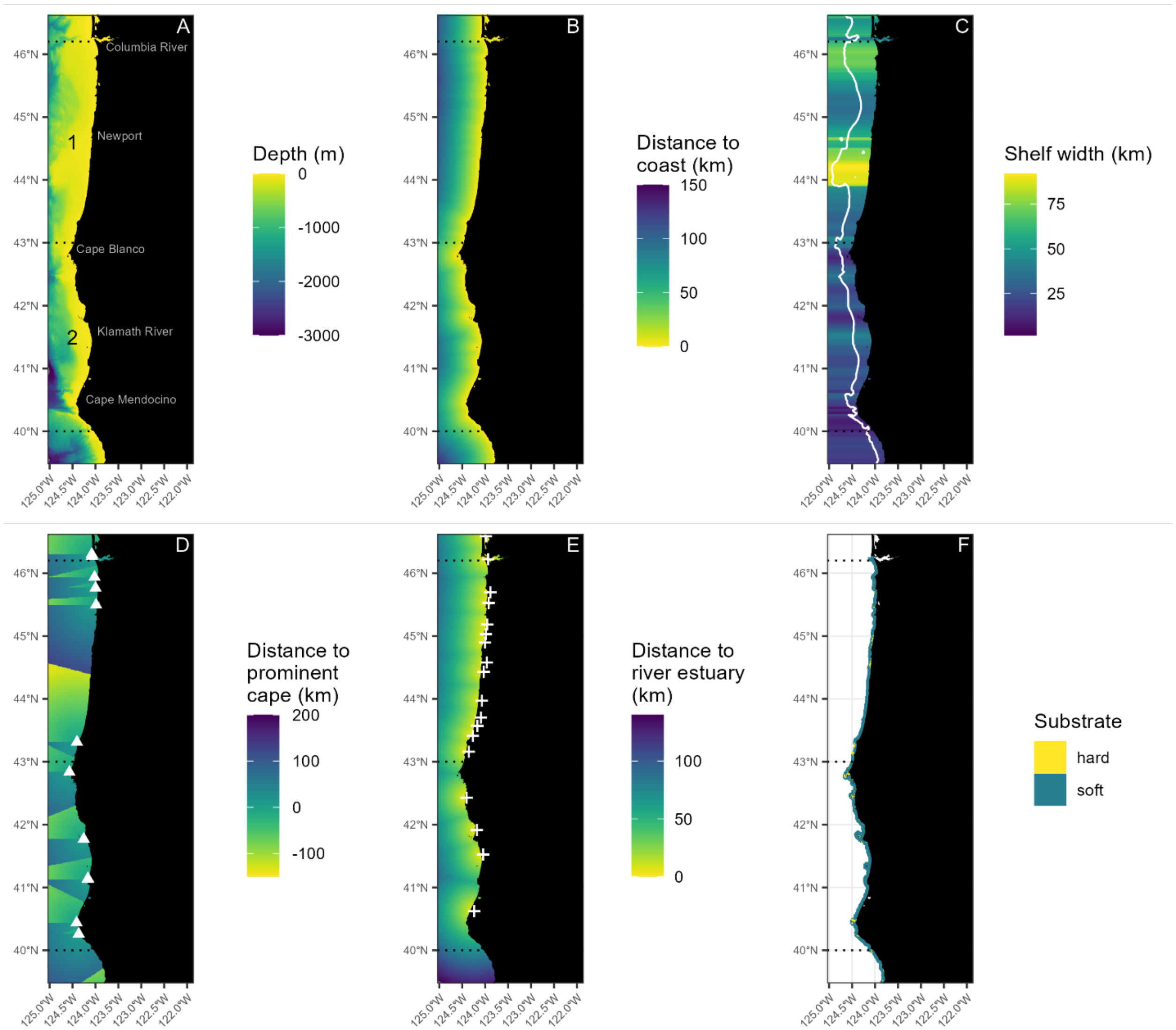

Figure S3. Static environmental predictors included in the gray whale density surface models: A) bathymetric depth, B) distance to the coast, C) shelf width, measured as the distance between the coast and the 200 m isobath, illustrated by the white line, D) distance to the nearest prominent cape, with negative values indicating distances north of the nearest cape, positive values indicating distances south of the nearest cape, and capes shown by the white triangles, E) distance to the nearest river estuary, with river estuaries show by the white crosses, and F) benthic substrate type.

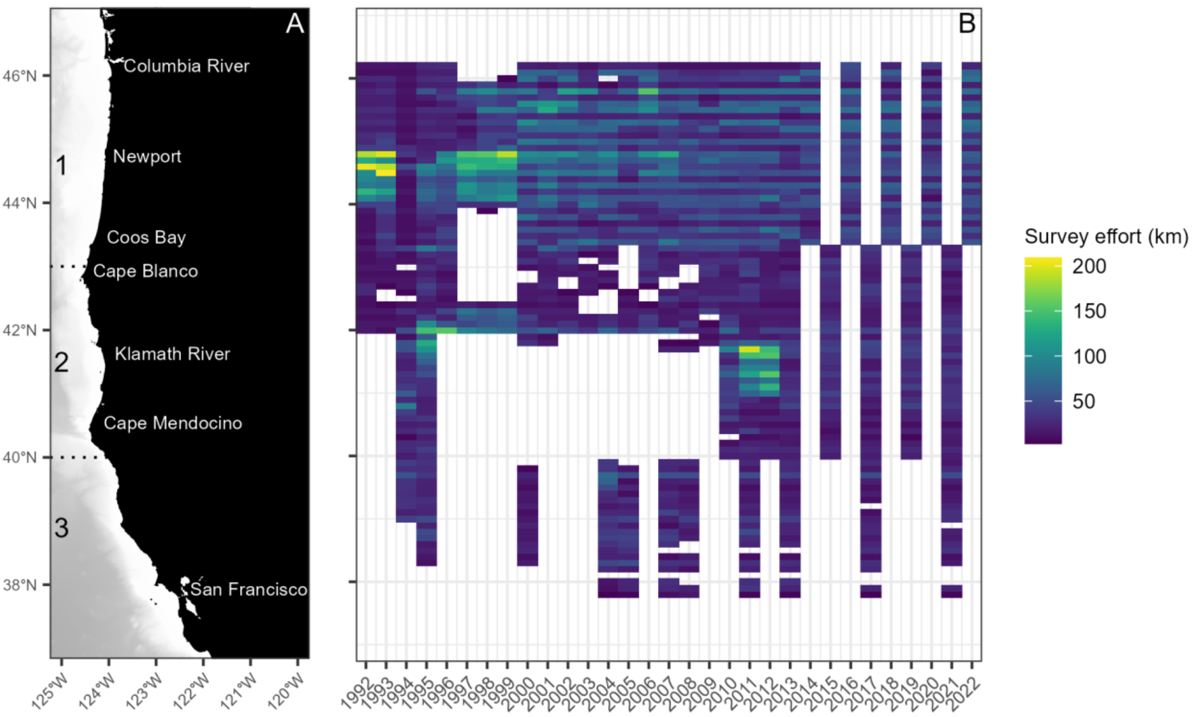

Figure S4. A) Map of the study area, with the region 1 and region 2 boundaries shown by the dashed lines, and major placenames denoted. B) Heatmap illustrating the survey effort throughout the study period, where the y-axis corresponds to the latitude on the map, the x-axis corresponds to the study year, and the fill color indicates the survey effort in km.

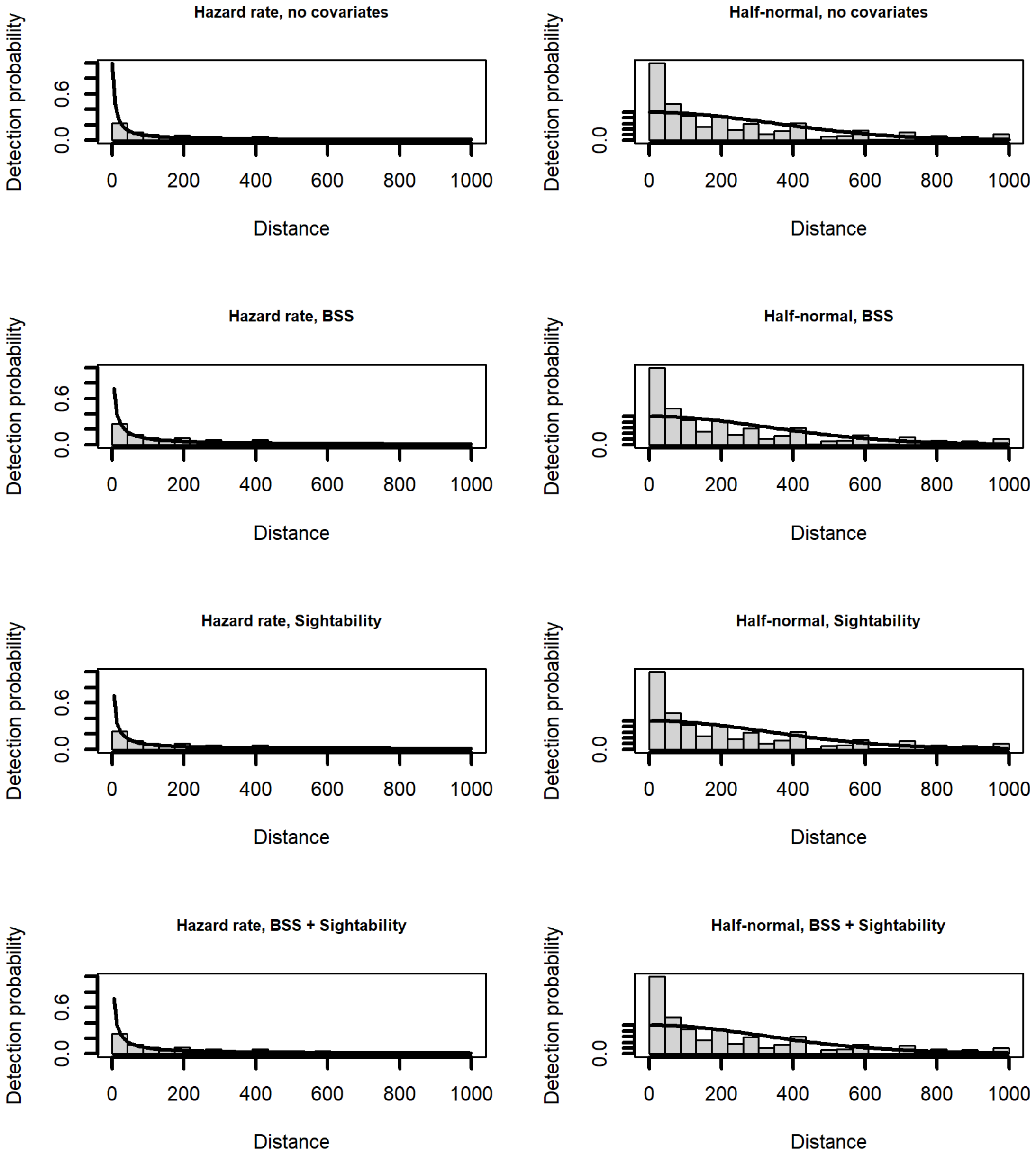

Figure S5. Candidate detection models fit with either a hazard rate (left column) or half-normal (left column) key, including no covariates (first row), Beaufort sea state (BSS, second row), sightability (third row), or both BSS and sightability (categorical classifier ranging from very bad to excellent, fourth row). The truncation distance has been set to the 98th percentile of the data, which is 1,000 m. Vertical bars show a histogram of the perpendicular distances between the gray whale observations and the survey trackline, and the black line shows the candidate detection function fit to the data, with detection probability shown on the y-axis. The selected detection function for the DSMs was fit with a half-normal key and BSS as a covariate. Corresponding comparisons of the candidate detection function performance are shown in Table S1.

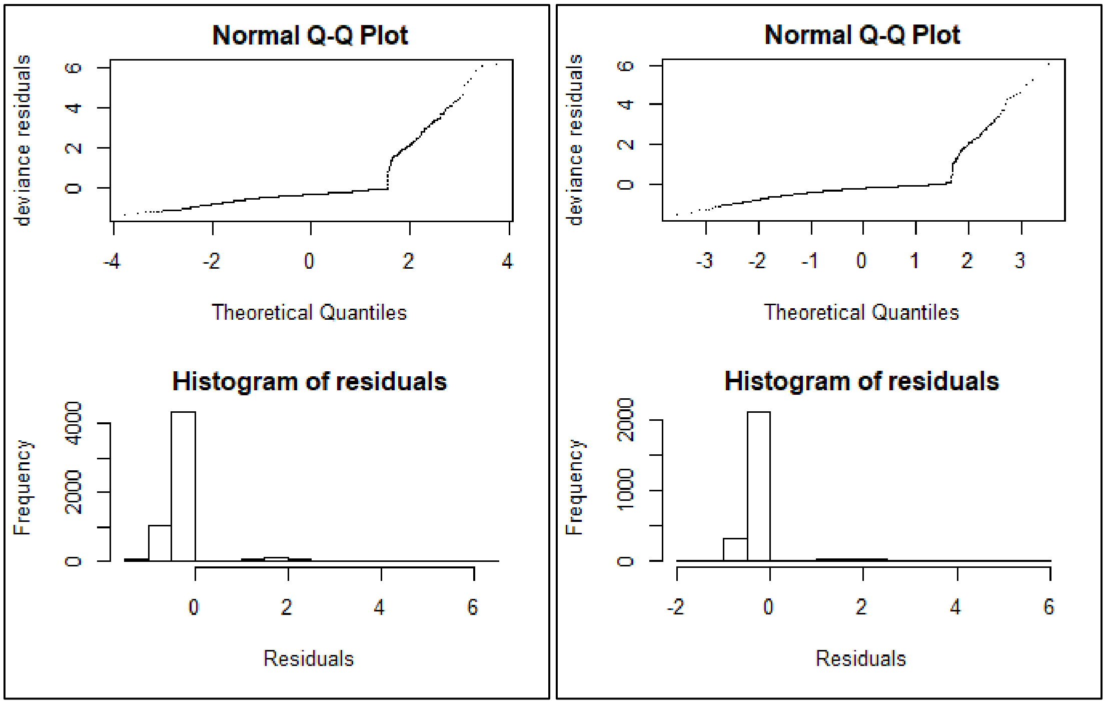

Figure S6. Diagnostic plots for GAM model outputs: normal Q-Q plots and histograms of residuals. The left column is for region 1, and the right column is for the region 2 model.

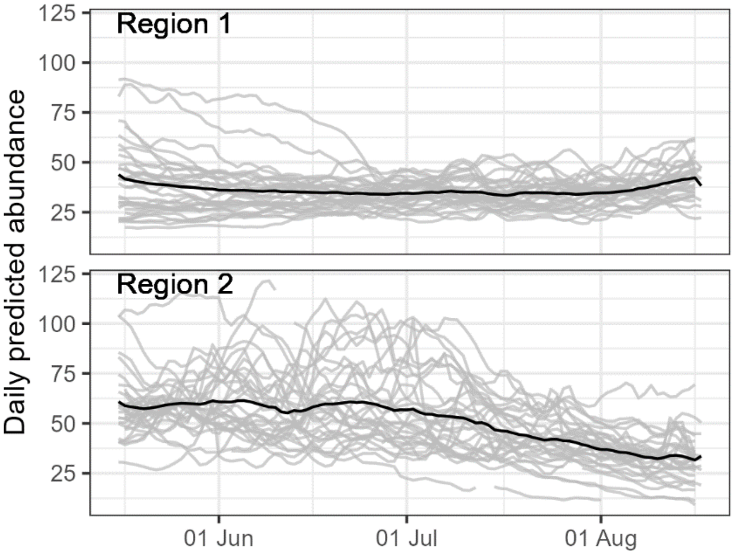

Figure S7. Daily predicted abundance estimates across the study period each year, for region 1 (top panel) and region 2 (bottom panel). Gray lines each represent one year, and the black line represents the mean of all years (1992-2022).

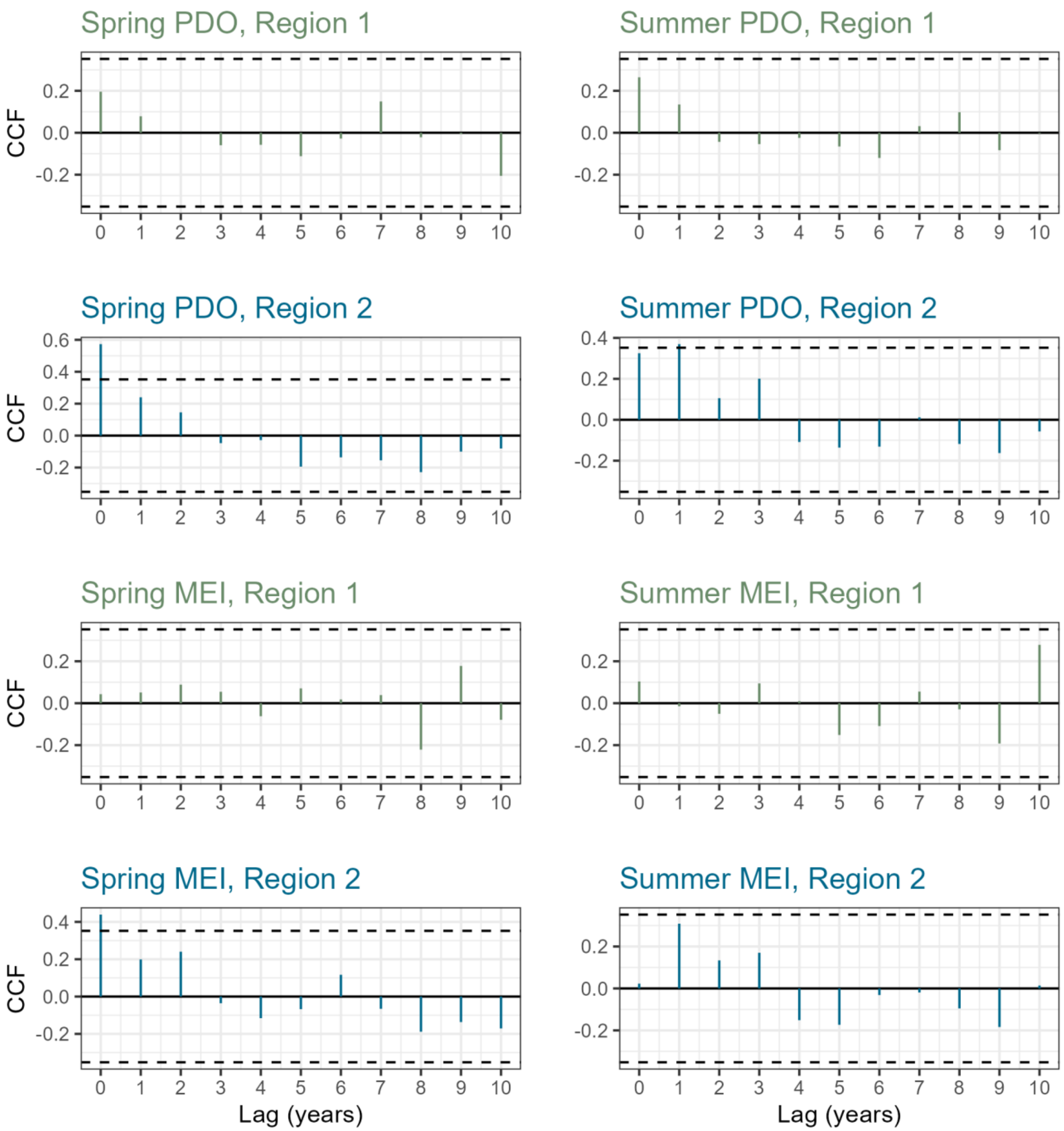

Figure S8. Timeseries cross-correlation results between predicted annual gray whale abundance and either the Pacific Decadal Oscillation (PDO) or Multivariate ENSO Index (MEI), in the spring (left column) or summer (right column), for each region.

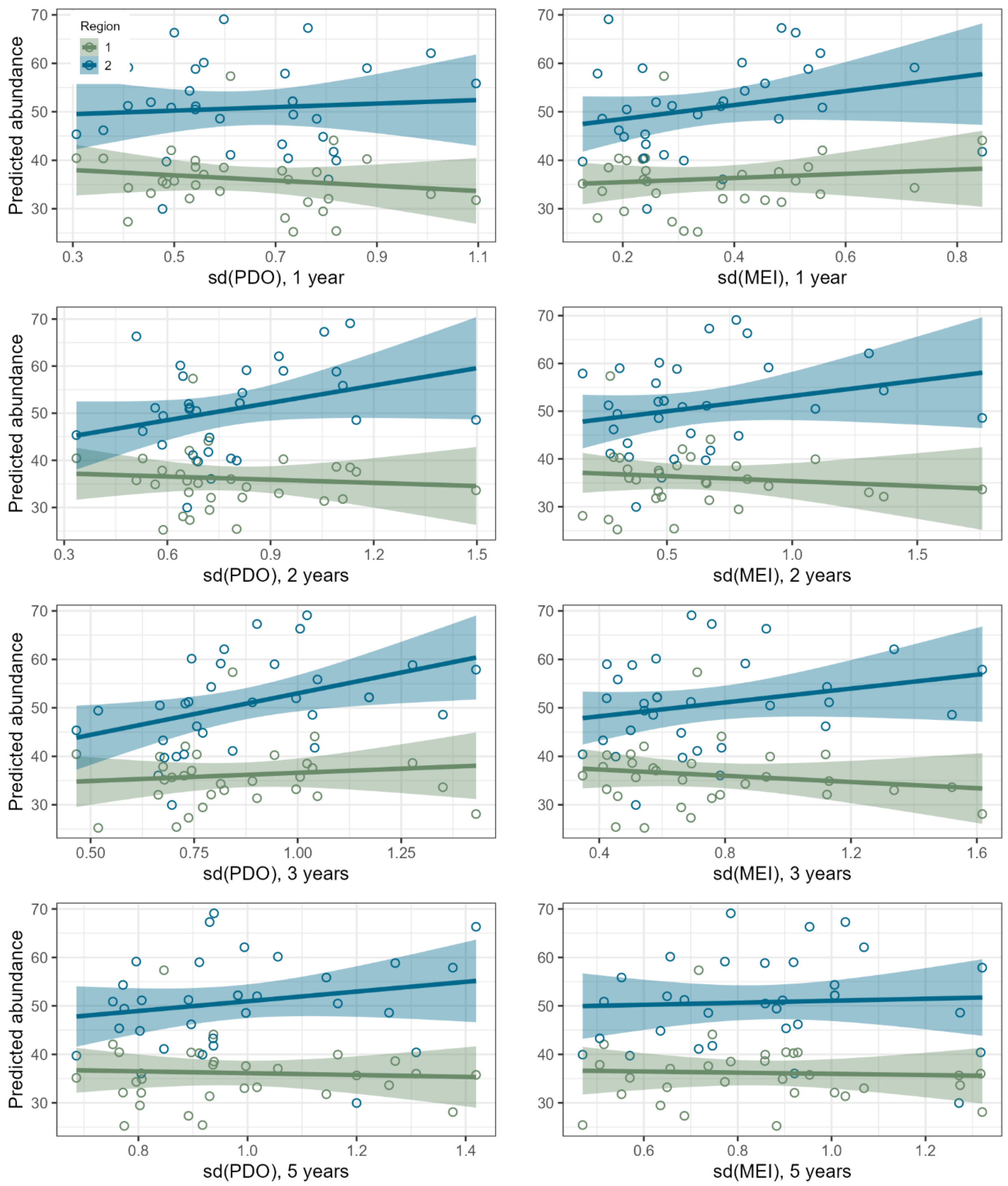

Figure S9. Relationship between predicted annual gray whale abundance and the variability in PDO and MEI, calculated over the preceding 1, 2, 3, and 5 years.

Table S1. Comparison of candidate detection function models, fitted with different covariates (none, Beaufort sea state, overall sightability) and different key functions (hazard-rate, half-normal). Models were compared using the difference in Akaike's information criterion (ΔAIC, lower value indicates better relative performance), and the realism of the predicted effective strip width (ESW) under different covariate conditions. In this case, the models that used a hazard-rate key produced unrealistically small ESW values despite having a lower AIC value, necessitating revised selection based on ecological knowledge of the system, as can be the case in cetacean distance sampling studies (Williams and Thomas 2007). Therefore, we elected to proceed with the best performing model that used a half-normal key, which included BSS as a covariate. Corresponding plots of the detection function fit to the distance data are shown for all candidate detection functions in Fig. S5.

**Covariates**	**Key function**	**ΔAIC**	**ESW range**	**Median ESW**
Sightability	Hazard-rate	0	28 - 458	34
BSS + Sightability	Hazard-rate	2.58	28 - 458	34
BSS	Hazard-rate	12.76	28 - 96	33
Null	Hazard-rate	14.93	34	34
BSS	Half-normal	274.48	323 - 530	432
BSS + Sightability	Half-normal	275.03	382-617	450
Sightability	Half-normal	277.46	382-617	450
Null	Half-normal	281.80	437	437

Table S2. Results of linear regression models evaluating the relationship between annual predicted gray whale abundance and variability in ocean basin-scale indices. Variability was calculated as the standard deviation of PDO and MEI, computed at four different timeframes: 1, 2, 3, and 5 years prior to the year of interest. Relationships were assessed separately for each region. Bold p-values with an asterisk (*) indicate a statistically significant linear relationship.

		Region 1			Region 2		
**Index**	**Timeframe**	R^2	$F_{(1,29)}$	**p**	R^2	$F_{(1,29)}$	**p**
sd(PDO)	1 year	0.021	0.648	0.427	0.005	0.152	0.698
	2 years	0.005	0.173	0.679	0.095	3.048	0.091
	3 years	0.012	0.373	0.546	0.171	6.003	**0.020***
	5 years	0.003	0.087	0.769	0.044	1.343	0.256
sd(MEI)	1 year	0.011	0.336	0.566	0.067	2.112	0.156
	2 years	0.011	0.338	0.564	0.059	1.820	0.187
	3 years	0.023	0.698	0.410	0.061	1.901	0.178
	5 years	0.001	0.051	0.822	0.002	0.079	0.779

References:

Schroeder, I. D., Santora, J. A., Mantua, N., Field, J. C., Wells, B. K., Hazen, E. L., ... & Bograd, S. J. (2022). Habitat compression indices for monitoring ocean conditions and ecosystem impacts within coastal upwelling systems. *Ecological Indicators*, *144*, 109520.

Williams, R., & Thomas, L. (2007). Distribution and abundance of marine mammals in the coastal waters of British Columbia, Canada. *J. Cetacean Res. Manage.*, *9*(1), 15-28.
